# The endogenous thrombin potential in patients with left ventricular assist device or heart transplant

**DOI:** 10.3389/fmed.2023.1155496

**Published:** 2023-04-12

**Authors:** Axel Schlagenhauf, Harald Haidl, Georg Trummer, Michael Berchtold-Herz, Jan-Steffen Pooth, Tanja Strini, Ulrich Geisen, Friedhelm Beyersdorf, Barbara Zieger

**Affiliations:** ^1^Department of Pediatrics and Adolescent Medicine, Division of General Pediatrics, Medical University Graz, Graz, Austria; ^2^Department of Cardiovascular Surgery, University Heart Center Freiburg-Bad Krozingen, Faculty of Medicine, University of Freiburg, Freiburg, Germany; ^3^Department of Emergency Medicine, Faculty of Medicine, Medical Center-University of Freiburg, Freiburg im Breisgau, Germany; ^4^Institute for Clinical Chemistry and Laboratory Medicine, Faculty of Medicine, University of Freiburg, Freiburg, Germany; ^5^Department of Pediatrics and Adolescent Medicine, Division of Pediatric Hematology and Oncology, Medical Center-University of Freiburg, Faculty of Medicine, University of Freiburg, Freiburg, Germany

**Keywords:** left ventricular assist device, Heartmate, coagulation, thrombin generation, bleeding

## Abstract

**Background:**

The Heartmate 3 (HM 3) is a left ventricular assist device featuring less shear stress, milder acquired von Willebrand syndrome, and fewer bleeding incidences than its predecessor the Heartmate II (HM II). The novel surface coating of the HM 3 suggests less contact activation of plasmatic coagulation. We hypothesized that patients with HM 3 exhibit fewer aberrations in their thrombin potential than patients with HM II. We compared these results with the thrombin potential of patients with heart transplantation (HTX).

**Methods:**

Thrombin generation in plasma samples of patients with HM II (*n* = 16), HM 3 (*n* = 20), and HTX (*n* = 13) was analyzed 3 days after implantation/transplantation and after long-term support (3–24 months) with HM II (*n* = 16) or HM 3 (*n* = 12) using calibrated automated thrombography. Heparin in postoperative samples was antagonized with polybrene.

**Results:**

Three days postoperatively HM II patients exhibited a lower endogenous thrombin potential (ETP) than HM 3 and HTX patients (HM II: 947 ± 291 nM*min; HM 3: 1231 ± 176 nM*min; HTX: 1376 ± 162 nM*min, *p* < 0.001) and a lower velocity index of thrombin generation (HM II: 18.74 ± 10.90 nM/min; HM 3: 32.41 ± 9.51 nM/min; HTX: 37.65 ± 9.41 nM/min, *p* < 0.01). Subtle differences in the thrombin generation profiles remained in HM II and HM 3 patients under long-term support (Velocity Index: HM II: 38.70 ± 28.46 nM/min; HM 3: 73.32 ± 32.83 nM/min, *p* < 0.05). Prothrombin fragments 1 + 2 were higher in HM II than in HM 3 patients (HM II: 377.7 ± 208.4 pM; HM 3: 202.1 ± 87.7 pM, *p* < 0.05) and correlated inversely with the ETP (r = −0.584, p < 0.05).

**Conclusion:**

We observed a more aberrant thrombin generation in HM II than in HM 3 despite comparable anticoagulation and routine parameters. A trend toward lower values was still observable in HM 3 compared to HTX patients. Calibrated automated thrombography may be a good tool to monitor the coagulation state of these patients and guide anticoagulation in the future.

## 1. Introduction

Patients with end-stage heart failure benefit from recent advances in the design of left ventricular assist devices (LVADs) which led to increased survival and improved quality of life. Smaller devices with less complications are not only implanted as bridge to transplant and bridge to recovery, but also favorable as destination therapy (1). Still, patients with LVAD exhibit substantially higher incidence rates of bleeding events as well as thromboembolic events which both can lead to fatalities (2, 3). Contact activation of the coagulation occurs on the surface of the devices, and anticoagulation is necessary to prevent systemic thrombosis and pump obstruction (4, 5). Additionally, abnormal hemodynamics lead to elevated shear stress which results in acquired von Willebrand syndrome (AVWS) and platelet dysfunction in VAD patients (6–8). Previously, we investigated AVWS and platelet secretion defects longitudinally in a large cohort of VAD patients (n = 198). We showed that AVWS develops rapidly upon LVAD implantation and disappeared upon explanation within hours (9). The degree of shear stress and the resulting severity of AVWS depends on the LVAD design. The Heartmate 3 (HM 3, Thoratec Corp) with a centrifugal flow design and artificial pulsatility resulted in an ameliorated degree of AVWS than the older continuous flow Heartmate II (HM II, Thoratec Corp) with axial flow design. Patients with HM 3 also had significantly fewer bleeding events compared to patients with HM II, most probably this may be attributed to design improvements (10). Nevertheless, all patients exhibited platelet ɑ- and δ-granule secretion defects independently of the LVAD design (9).

Another aspect not yet well understood is the degree of contact activation of the plasmatic coagulation system in LVAD patients. In contrast to normal hemostasis which is triggered upon vessel damage by tissue factor expression, contact activation is a non-physiological activation of factor XII on foreign surfaces which is strongly associated with thrombosis (11). Resulting thrombin generation can lead to increased levels of thrombin/antithrombin complexes, prothrombin fragments 1 and 2 (F1 + 2), and D-dimers in LVAD patients (12). The HM 3 surfaces are textured with titanium microspheres to stimulate formation of an endothelial coating, which might improve hemocompatibility and reduce contact activation (10, 13). However, acute pump thrombosis can occur also with HM 3 at low-flow and subtherapeutic levels of anticoagulation (14). Continuous subclinical contact activation may also lead to clotting factor consumption which adds to increased bleeding risk caused by AVWS and platelet dysfunction.

While global tests of coagulation, such as INR (Quick) and aPTT, are not sensitive enough to detect subtle changes in coagulation, the continuous tracing of thrombin over time can expose even minor shifts in the hemostatic balance (15). Calibrated automated thrombography enables the time-dependent tracing of thrombin *in-vitro* using a fluorescent substrate. The overall amount of thrombin that can be generated is represented by the endogenous thrombin potential (ETP) which reflects the overall balance of the hemostatic system. The ETP is sensitive to states of hypo- and hypercoagulability, clotting factor deficiencies, and anticoagulation (16–18).

We hypothesized that patients with HM II, HM 3, and heart transplants (HTX) exhibit varying degrees of continuous contact activation, and that potential clotting factor deficiencies would be reflected by differences in the patients’ ETP. Aim of this study was to investigate thrombin generation using calibrated automated thrombography in patients with HM II or HM 3 postoperatively with *in-vitro* reversal of heparin to depict the actual state of the hemostatic system without anticoagulation. Furthermore, these results were compared with data obtained from HTX patients Additionally, we aimed to evaluate the long-term effects of LVAD implantation on thrombin generation in patients with HM II, HM 3, and HTX receiving phenprocoumon.

## 2. Materials and methods

### 2.1. Patients

In total, 77 patients who received an LVAD or heart transplant between 2013 and 2016 at the University Hospital in Freiburg were included in this study. From 49 patients samples were taken on day 3 after implantation/transplantation (HM II: *n* = 16, HM 3: *n* = 20, HTX: *n* = 13). From 28 patients samples were taken 3 months to 2 years after implantation/transplantation (HM II: *n* = 16, HM 3: *n* = 12). Patients were recruited within the scope of our institutional monitoring program on hemostaseological changes during support *via* VAD, approved by the ethics committee of the University of Freiburg and supported by the German Research Foundation. All patients provided their informed consent.

### 2.2. Surgical procedures

LVADs were implanted according to techniques previously described (19, 20). The inflow graft was inserted into the left ventricular apex and the outflow graft anastomosed to the ascending aorta (21, 22). The HM II was typically operating at 8.600–10.400 rpm; the HM 3 at 5.000–6.000 rpm. Pump speed for initial operation during the implant procedure was determined by transesophageal echocardiography to minimize septical shifting. This procedure was used routinely in both HM II and HM 3 pumps and was also used for pump speed adaption during the first postoperative days. To achieve these optimal settings regarding ventricular geometry, different ranges of pump speed in HM II vs. HM 3 pumps are required, caused by the different techniques of both pumps.

Anticoagulation was started with heparin after 48 h in all LVAD patients with a target activated partial thromboplastin time (aPTT) of 60–80 s. In addition, the heparin effect was measured by anti-Xa (target 0.2 IU/mL). Phenprocoumon was initiated after removal of the chest drains and sufficient oral ingestion.[22] The target international normalized ratio (INR) was 2.0–3.0. Acetylsalicylic acid (ASA) 100 mg/day was used in addition to inhibit platelet aggregation when INR was stable at the target level. The effect of ASA was monitored by aggregometry analyses.

Heart transplantation was performed with bicaval or biatrial anastomosis of the donor’s heart. Patients received low-dose heparin (250–500 IU/h) and ASA 100 mg/day postoperatively.

### 2.3. Laboratory experiments

Thrombin generation was performed using Calibrated Automated Thrombography according to Hemker et al. as reported previously (16). This method allows continuous tracing of thrombin generated *in-vitro* in a specific plasma sample over time. Thrombin generation assays were performed using a Fluoroskan Ascent plate reader (Thermo Labsystems, Helsinki, Finland) and Thrombinoscope© software (Thrombinoscope BV, Maastricht, the Netherlands). Twenty microliter of PPP-reagent containing 4 μM phospholipids and tissue factor (5 pM or 20 pM final concentration) or calibrator (both Thrombinoscope BV, Maastricht, The Netherlands) were placed into respective wells of a 96-well-plate (Immulon 2 HB, Thermo Scientific). Eighty microliter plasma was added. The measurement was started by automatic dispensing of 20 μl fluobuffer-CaCl2 (20 mM Hepes, 140 mM NaCl, 5 mg/mL bovine serum albumin, and 16.7 mM CaCl2 final concentration, pH 7.35), containing a fluorogenic substrate (Z-Gly-Gly-Arg-amino-methyl-coumarin, Bachem, Bubendorf, 417 μM final concentration). Thrombin generation profiles were recorded in triplicates.

**Figure 1:**
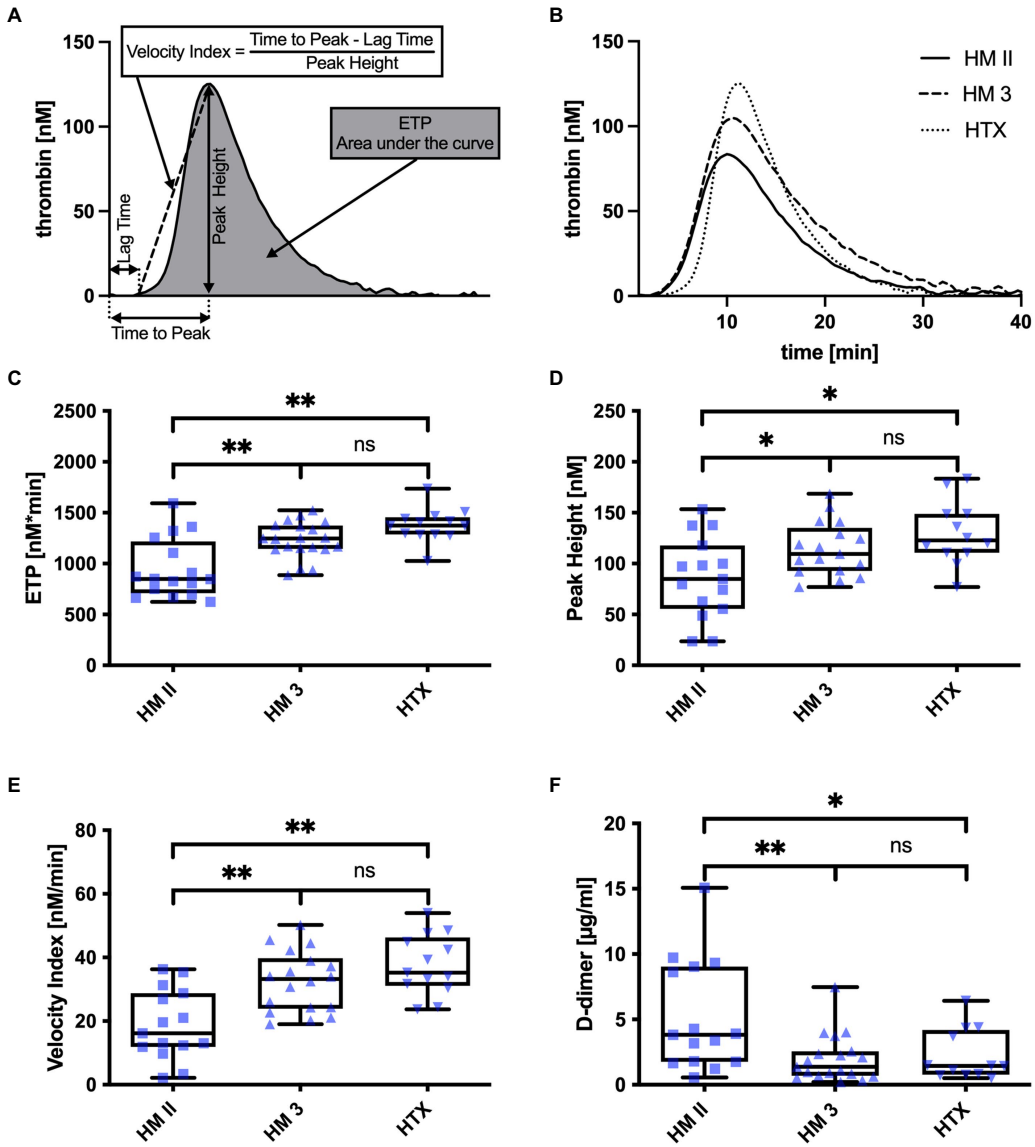
**(A)** Schematics of a thrombin generation trace and deduced parameters; **(B)** Representative thrombin generation traces of a patient with HM II, HM 3, and HTX; **(C)** ETP, **(D)** Peak Height, **(E)** Velocity Index, and **(F)** D-Dimer values of patients with HM II, HM 3, and HTX. *p < 0.05; **p < 0.001.

The effect of heparin in samples from patients on day 3 after implantation/transplantation was antagonized with polybrene (0.03 mg/mL final concentration) and thrombin generation was triggered with 5 pM tissue factor (final concentration). Samples from patients with long-term implantation/transplantation receiving phenprocoumon were measured without polybrene and 20 pM tissue factor (final concentration). The parameters derived from the thrombin generation trace are schematically depicted in Figure 1A.

Platelet counts, INR, aPTT, and values for factor VIII- and XIII-activity, fibrinogen, antithrombin, D-dimer, and VWF antigen were determined using routine laboratory methods. Prothrombin fragments 1 + 2 (F1 + 2) were determined with ELISA (Siemens Healthcare Diagnostics, Erlangen, Germany).

### 2.4. Statistics

Values for age and routine coagulation parameters are depicted as mean ± standard deviation. Distributions of thrombin generation parameters are depicted in box-and-whisker plots. Differences between HM II, HM 3, and HTX were analyzed using ANOVA. Corrections for multiple comparisons were made *via* the Holm-Šídák method (a = 0.05), and multiplicity-adjusted *p*-values were calculated for each comparison. Correlations between parameters were calculated using Pearson correlation analysis. Calculations were done using Graphpad Prism 9.0 (Graphpad Software, San Diego, CA) and IBM SPSS Statistics Version 24 (IBM Corp., Armonk, NY, United States).

## 3. Results

### 3.1. Demographical data

The patients recruited 3 days postoperatively were under HM II or HM 3 support or received HTX. The HM II cohort (*n* = 16) had a median age of 46 years (range: 25–74 years) and consisted of five female and 11 male patients. The HM 3 cohort (*n* = 20) had a median age of 59 years (range: 35–73 years) and consisted of one female and 19 male patients. The HTX cohort (*n* = 13) had a median age of 52 years (range: 27–61 years) and consisted of three female and 10 male patients. Out of the 13 patients in the HTX cohort, seven required mechanical circulatory support prior to transplantation. Of these, two were supported by an HM II, two by an HM II with RVAD, and one by an HM 3 with RVAD. All patients received antithrombotic treatment with heparin.

The patients recruited 3 to 24 months after LVAD implantation were under HM II or HM 3 support. The HM II cohort (*n* = 16) had a median age of 50 years (range: 21–70 years) and consisted of one female and 15 male patients. The HM 3 cohort (*n* = 12) had a median age of 55 years (range: 44–65 years) and consisted of 12 male patients. All patients received antithrombotic treatment with phenprocoumon.

### 3.2. Thrombin generation parameters

#### 3.2.1. Postoperatively

Heparin in plasma samples from patients on day 3 after LVAD implantation or heart transplantation were antagonized *in-vitro* with polybrene before measurement with calibrated automated thrombography. Representative thrombin generation traces are depicted in (Figure 1B). ANOVA of thrombin generation parameters showed differences in the endogenous thrombin potential (HM II: 947 ± 291 nM*min; HM 3: 1231 ± 176 nM*min; HTX: 1376 ± 162 nM*min, *p* < 0.001) (Figure 1C), the peak height (HM II: 86.35 ± 39.79 nM; HM 3: 114.4 ± 26.3 nM; HTX: 129.7 ± 31.1 nM, *p* < 0.05) (Figure 1D), and the velocity index (HM II: 18.74 ± 10.90 nM/min; HM 3: 32.41 ± 9.51 nM/min; HTX: 37.65 ± 9.41 nM/min, p < 0.001) (Figure 1E). Analysis of these parameters revealed significant differences between HM II and HTX patients, but not between HM 3 and HTX patients.

#### 3.2.2. On long-term support

**Figure 2:**
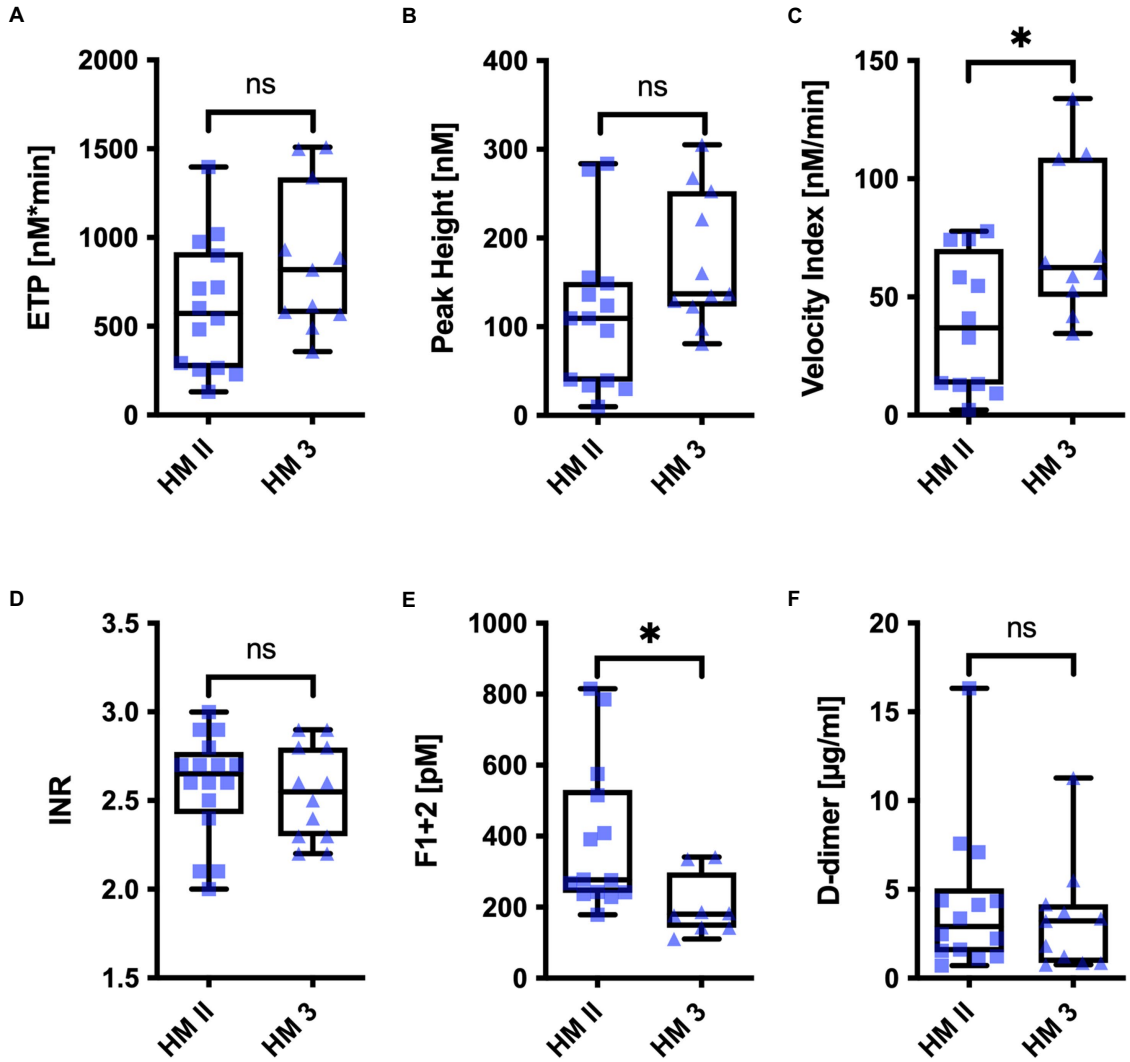
**(A)** ETP, **(B)** Peak Height, **(C)** Velocity Index, **(D)** INR, **(E)** Prothrombin Fragments 1 and 2, and **(F)** D-Dimer of patients with HM II, and HM 3 on long-term support. *p < 0.05.

Differences were less pronounced but still observable in patients under long-term HM II or HM 3 support receiving phenprocoumon (Figure 2). Despite comparable INR, the Velocity Index was significantly lower in HM II patients compared to HM 3 patients (HM II: 38.70 ± 28.46 nM/min; HM 3: 73.32 ± 32.83 nM/min, *p* < 0.05) (Figure 2C).

### 3.3. Routine coagulation markers and bleeding events

Routine markers of coagulation on day 3 after implantation/transplantation are listed in Table 1. Patients showed increased levels of acute phase proteins (Factor VIII, VWF, fibrinogen). D-dimer levels were pathologically elevated in all patients secondary to the surgery, but HM II patients showed significantly higher levels than HM 3 and HTX patients (*p* < 0.05) (Figure 1F). APTT was prolonged in all patients, but it was more prolonged in HM II compared to HM 3 patients (*p* < 0.05). HM 3 patients showed slightly lower platelet counts than HM II patients (p < 0.05), but none of the patients exhibited pronounced thrombocytopenia. Antithrombin levels were lower in HM II compared to HTX patients (*p* < 0.05).

**Table 1:** Routine coagulation of HM II (*n* = 16), HM 3 (*n* = 20), and HTX patients (*n* = 13) postoperatively.

Parameter	HM II	HM 3	HTX	Reference range
INR	1.10 ± 0.13	1.32 ± 0.42	1.77 ± 0.95	0.85–1.15
aPTT [s]	65.0 ± 22.7	51.1 ± 9.4^§^	52.2 ± 16.6	25.1–37.7
Platelet count [*10^9^/L]	250 ± 130	154 ± 73^§^	194 ± 112	146-350
Factor VIII [%]	267 ± 48	328 ± 72	318 ± 89	60–180
Factor XIII [%]	78 ± 18	86 ± 28	94 ± 20	62–150
Fibrinogen [mg/dL]	475 ± 275	655 ± 218	486 ± 107	170–420
Antithrombin [%]	73 ± 15*	84 ± 21	92 ± 19	50–170
VWF:Ag [%]	312 ± 115	382 ± 93	283 ± 123	60–150
D-dimer [μg/mL]	5.16 ± 4.20	1.99 ± 1.80	2.27 ± 1.94	<0.5

§ Statistically different from HM II.

^*^Statistically different from HTX.

Routine markers of coagulation 3 months to 2 years after implantation are listed in Table 2. None of these markers differed significantly between HM II and HM 3 patients. However, HM II patients showed slightly higher F1 + 2 levels than HM 3 patients (HM II: 377.7 ± 208.4 pM; HM 3: 202.1 ± 87.7 pM, p < 0.05) (Figure 2E). Interestingly, there was an inverse correlation observable between F1 + 2 levels and the ETP in thrombin generation in the patients with HM II or HM 3 (r = −0.584, p < 0.05).

**Table 2:** Routine coagulation of HM II (*n* = 16), HM 3 (*n* = 12) patients on long-term support.

Parameter	HM II	HM 3	Reference range
INR	2.58 ± 0.30	2.54 ± 0.26	0.85–1.15
aPTT [s]	53.5 ± 12.9	51.7 ± 10.7	25.1–37.7
Factor VIII [%]	264 ± 95	275 ± 99	60–180
Factor XIII [%]	95 ± 28	88 ± 33	62–150
Fibrinogen [mg/dL]	416 ± 160	398 ± 111	170–420
Antithrombin [%]	89 ± 19	80 ± 23	80–130
D-dimer [μg/mL]	4.15 ± 4.11	3.34 ± 3.07	<0.5

We monitored bleeding incidence within 30 days after implantation/transplantation (Table 3). We observed fewer incidences of bleeding in patients with HM 3 compared to HM II, particularly within the first 30 days. Our findings showed that the incidence within 30 days was substantially higher in the HM II patients, while the postoperative bleeding incidence (within 7 days) was similar between HM II and HM 3 patients. Interestingly, while the number of patients exhibiting hemorrhages was comparable in both cohorts, HM 3 patients only experienced singular bleeding events: whereas five out of seven patients with hemorrhages in the HM II cohort had repeated bleeding events, resulting in a significantly higher event rate per patient-months. As hypothesized, both LVADs were associated with higher event rates compared to HTX. Although these trends were clearly observable, the cohorts were too small for statistically valid analyses.

**Table 3:** Number of bleeding complications and number of patients with respective bleeding complications (parenthesized) within seven and 30 days after surgery in HM II, HM 3, and HTX cohorts.

Parameter	Within 7 days	Within 30 days	Event rate (per patient-months)
HM II (*n* = 20)	5 (3)	14 (7)	0.70
HM 3 (*n* = 16)	4 (4)	6 (6)	0.38
HTX (*n* = 13)	0 (0)	1 (1)	0.08

## 4. Discussion

The impact of LVAD implantation on patients’ plasmatic coagulation system is challenging to investigate due to additional altering factors, particularly surgery itself and required anticoagulation (23). Thrombin generation before and after LVAD implantation has been examined in a recent study (24). However, the authors focused on longitudinal progression and did not antagonize heparinized samples. To our knowledge, this is the first study comparing thrombin generation in HM II and HM 3 patients.

We focused on potential contact activation on the LVAD surface which may lead to moderate clotting factor consumption, thus, adding to the bleeding risk. Hence, we first chose to investigate thrombin generation on day 3 after implantation or transplantation, because plasma of patients with heparin prophylaxis could be antagonized *in-vitro* with polybrene which was not possible later on when patients received phenprocoumon. Heparinization was monitored by anti-factor Xa activity.

In the antagonized samples we observed differences in the endogenous thrombin potential, the peak height, and the velocity index. Patients with HM 3 exhibited fewer alterations than patients with HM II. Consequently, thrombin generation profiles of HM II patients differed significantly from those of HTX patients. A trend without statistically significance toward lower thrombin generation parameters was still observable in HM 3 compared to HTX patients (Figure 1). The higher ETP in HM 3 compared to HM II patients may be attributable to design improvements resulting in a lower degree of contact activation in the newer device (9).

Some HTX patients required mechanical circulatory support prior to transplantation. However, we did not observe significant differences in coagulation parameters or thrombin generation between patients with or without prior mechanical circulatory support. Moreover, the only bleeding event in the HTX cohort occurred in a patient without previous mechanical circulatory support. This finding is consistent with our previous research indicating that the hemodynamic effects of mechanical circulatory support on coagulation typically subside within 24 h following explanation (9, 25).

We also observed subtle differences regarding thrombin generation in samples from HM II and HM 3 patients several months after implantation when the acute phase response had subsided. The values for the Velocity Index in HM 3 were still significantly higher compared to the values in HM II patients. These patients received phenprocoumon, and we acknowledge that slight differences in anticoagulation could be a potential bias. However, INR was comparable between these cohorts. More importantly, differences in ETP coincided with increased F1 + 2 in the HM II cohort. Potentially, INR is not sensitive enough to detect subtle changes attributable to moderate clotting factor consumption. Interestingly, ETP correlated inversely with F1 + 2 in these cohorts, again hinting at mild consumption.

In our setting, the ETP provides insight into the clotting factor interplay. HM 3 and HTX patients exhibited fewer alterations indicating a higher robustness of the hemostatic balance than HM II patients.

This finding is consistent with the reduced bleeding incidence that we observed in HM 3 patients compared to HM II patients, particularly after the postoperative acute phase response had subsided (Table 3). Our previous large cohort study on acquired von Willebrand syndrome yielded similar results, further supporting the better clinical outcome in HM 3 patients (9). While we did not systematically monitor thromboembolic events, we did observe significantly fewer events after migration to HM 3 as first-line therapy.

### 4.1. Limitations

A limitation of measuring postoperative samples may be that patients are potentially still in the acute phase response following surgical trauma. Thrombin generation markers (e.g., D-dimer) are still increased due to the procedure. On the other hand, we gained a snapshot of the coagulation system unbiased by anticoagulation and a comparable time-point for all three cohorts. Patients on long-term support could not be compared with HTX patients, because the latter do not receive anticoagulation with phenprocoumon. Nevertheless, the observed differences between HM II and HM 3 patients were consistently observable and are in line with the clinical findings of our previous study (9).

### 4.2. Conclusion

Taken together, we observed differences in thrombin generation profiles of patients with HM II, HM 3, and HTX despite comparable anticoagulation and routine parameters. Patients with HM II exhibited lower ETP, lower peak height, and lower velocity index than patients with HM 3 or HTX. Therefore, calibrated automated thrombography seems to be a good tool to monitor the coagulation state of these patients because of the high sensitivity. This may be particularly useful because complete omittance of anticoagulation is currently being discussed for newer LVADs to decrease the bleeding risk (26). Thrombosis, contact activation, and resulting clotting factor consumption remain to be an issue with the current devices. Cautious weaning of anticoagulation may be feasible on an individual basis with appropriate methods to monitor coagulation.

## Data availability statement

The raw data supporting the conclusions of this article will be made available by the authors, without undue reservation.

## Ethics statement

The studies involving human participants were reviewed and approved by Ethics Committee of the University of Freiburg. The patients/participants provided their written informed consent to participate in this study.

## Author contributions

AS: conceptualization, methodology, investigation, formal analysis, validation, original draft preparation, and review and editing. HH: validation, original draft preparation, and review and editing. GT: clinical care for the patient, validation, and review and editing. MB-H: clinical care for the patient, validation, review, and editing. J-SP: validation, review, and editing. TS: methodology, investigation, and validation. UG: methodology, formal analysis review, and editing FB: validation, review, and editing. BZ: conceptualization, validation, investigation, and review and editing. All authors contributed to the article and approved the submitted version.

## Funding

This work was supported by the German Research Foundation (DFG), Bonn, Germany, as part of the comprehensive project “Cardiac assist devices for long-term support in cardiac insufficiency” (reference no. PAK 350, ZI486/7–1).

## Conflict of interest

The authors declare that the research was conducted in the absence of any commercial or financial relationships that could be construed as a potential conflict of interest.

## Publisher’s note

All claims expressed in this article are solely those of the authors and do not necessarily represent those of their affiliated organizations, or those of the publisher, the editors and the reviewers. Any product that may be evaluated in this article, or claim that may be made by its manufacturer, is not guaranteed or endorsed by the publisher.
